# Walking aids and complicated orthopedic diseases are risk factors for falls in hemodialysis patients: an observational study

**DOI:** 10.1186/s12877-023-04015-9

**Published:** 2023-05-22

**Authors:** Takeo Ishii, Wataru Matsumoto, Yui Hoshino, Yasuhiro Kagawa, Emi Iwasaki, Hiromi Takada, Takashi Honma, Kunio Oyama

**Affiliations:** 1 Yokohama Dai-Ichi Hospital, Internal Medicine, 2-5-15 Takashima, Nishi-Ku, Yokohama, Kanagawa 220-0011 Japan; 2 Genkikai Yokohama Hospital, Yokohama, 226-0013 Japan; 3 Tama-Mukogaoka Jin Clinic, 4Th Floor, Noborito Building, 1780 Noborito, Tama-Ku, Kawasaki, Kanagawa 214-0014 Japan; 4 Hachioji Jin Clinic, Southern Sky Tower Hachioji 3Rd Floor, 4-7-1 Koyasumachi, Hachioji, Tokyo 192-0904 Japan; 5 Yokohama Higashiguchi Jin Clinic, 6Th Floor, Yokohama Station Square Common Building ,2-13-2 Takashima, Nishi-Ku, Yokohama, Kanagawa 220-0011 Japan; 6 ZENJINKAI Medical Corporation, F6, 2-5-12 Takashima, Nishi-Ku, Yokohama, Kanagawa 220-0011 Japan

**Keywords:** Hemodialysis, Fall risk, Early assessment, End-stage renal disease

## Abstract

**Background:**

Aging and an increased fall risk have been demonstrated in hemodialysis patients at home and in a facility. However, studies investigating the cause of falls to prevent fractures in dialysis rooms are scarce. This study aimed to explore the related factors for accidental falls statistically in dialysis facilities for future fall prevention.

**Methods:**

This study included 629 hemodialysis patients with end-stage renal disease. The patients were divided into two groups: the fall and non-fall groups. The main outcome was the presence or absence of falls in the dialysis room. Univariate and multivariate logistic analyses were performed; multivariate analysis was conducted using covariates significantly correlated in the univariate analysis.

**Results:**

A total of 133 patients experienced falling accidents during the study period. The multivariate analysis indicated that the use of walking aid (*p* < 0.001), orthopedic diseases (*p* < 0.05), cerebrovascular disease, and age were significantly correlated with falls.

**Conclusions:**

In the dialysis clinic, patients who use walking aids and have complicated orthopedic or cerebrovascular conditions are at a high risk of falling in the dialysis room. Therefore, establishing a safe environment may help prevent falls, not only for these patients but also among other patients with similar conditions.

## Background

The hemodialysis (HD) population is aging [1, 2]. Older HD patients are at a higher risk of falling due to frailty, hypotension [3], sarcopenia owing to chronic kidney disease [4], and others. Some studies have shown that risk factors for falls include age, comorbidities, mean pre-dialysis systolic blood pressure, history of falls, muscle weakness, gait deficits, balance deficits, the use of assistive devices, visual deficits, arthritis, impaired activities of daily living (ADL), depression, and cognitive impairment [5–10]. In a study by Cook et al., patients were monitored for accidental falls using biweekly interviews in the HD unit [5], which included incidents that occurred outside the dialysis room and during non-dialysis days. Conversely, our facilities had safety teams consisting of clinical engineers and nurses assigned to each unit who reported falls occurring in the dialysis facility. Although limited in terms of time and space, this approach may allowed for a more precise investigation of cause-and-effect relationships.

Generally, older patients undergoing dialysis have a high incidence of falls and a high fracture rate [11]. Given the high complication rate, older patients at risk of falling should be identified and managed [12]. The risk of hip fracture was fivefold higher in dialysis patients than in the general population [13]. Moreover, fractures due to falling are increasingly recognized as risk factors for other poor outcomes such as hospitalization and death [2, 14–17].

One study has shown that one accidental fall in a community of HD patients aged ≥ 65 years was associated with an increased independent risk of death [18]. Another study has suggested that the prevention of each serious fall would have resulted in cost savings between $25,158 and $36,781 [19]. Previous studies have shown that decreased limb strength, poor physical performance, and compromised physical functions such as balance are critical risk factors for falls [20]. Exercise, such as strength and balance training, and tai chi, have been suggested as measures to prevent falls [4, 21–23]. However, such exercise interventions to prevent falls in long-term care facilities have been shown to be beneficial only when performed for at least 6 months or longer [24]. Additionally, exercise therapy requires specialized staff to maintain its effectiveness and safety. The rate of falls substantially increases in the first month after hospitalization [25]. Patients with a history of falls 6 months before admission are twice as likely to fall within 3 months of discharge [26], which is earlier than when exercise therapy is typically expected to be effective. Environmental changes can also increase the risk of falls, as seen in dialysis patients who had a higher fall rate when discharged from the hospital following the introduction of dialysis in a new environment. Therefore, it is imperative to implement fall prevention measures at the onset of hospital visits to dialysis facilities. Reducing the risk of falls in the months leading up to physical function improvement is crucial.

Falls that occur within the hospital setting can result in serious injuries, leading to a rise in hospital costs and extended hospital stays [27]. Therefore, it is critical to prevent falls in dialysis room to maintain the patients’ autonomy at home. Moreover, there is limited research regarding the cause of falls to prevent fractures in dialysis rooms. Therefore, we aimed to investigate the causes of falls in the dialysis room and suggest preventive measures.

## Methods

### Medical safety teams

To enhance the safety of dialysis patients and assure their well-being, our group clinics have established medical safety teams comprising clinical engineers and nurses, which were assigned to each facility and reported falls or other safety concerns occurring in the dialysis facility. The medical safety teams discussed the reports on a regular basis and attempted to implement the necessary measures to improve patient safety in each facility.

### Study participants

This study involved 629 patients with end-stage renal disease who underwent HD treatment between April 2016 and March 2018 at 8 dialysis centers located in Yokohama, Kanagawa, Japan (Table 1). The main outcome was the presence or absence of a fall in a facility as reported by the medical safety teams. A fall was defined as an unintentional movement resulting in the patient coming to rest on the floor or ground [28] in the dialysis facility room. The study was approved by the ZENJINKAI ethics committee (project number 2018-0003) and carried out in accordance with the Declaration of Helsinki. Informed consent was obtained in the form of opt-out on the website. Those who declined were excluded from the study. Since the data were anonymized, the need for written informed consent was waived. The Ethics Committee of Zenjinkai Yokohama Daiichi Hospital approved this study (Approval number: 20200723).

**Table 1:** Patient characteristics

	All	Falls (*n* = 133)	Non-falls (*n* = 496)	*P*-value
**Baseline**				
Age (years)	68.7 ± 12.4	73.8 ± (11.2)	67.2 ± 12.4	< 0.001
Sex (male/female)	412/350 (65.5)	331/298 (66.7)	81/52 (60.9)	0.483
Height (cm)	161.1 ± 11.4	159.4 ± 9.8	161.9 ± 9.2	0.006
DW (kg)	58 ± 14.1	55.3 ± 13.1	58.5 ± 13.8	0.016
BMI (kg/m^2^)	21.9 ± 3.9	21.6 ± 3.9	22.1 ± 3.9	0.197
**Complications**				
Orthopedic diseases (%)	199 (31.6)	66/133 (49.6)	133/496 (26.8)	< 0.001
Heart diseases (%)	260 (413)	63/133 (47.4)	196/496 (39.5)	0.113
Cerebrovascular diseases (%)	116 (18.4)	39/133 (29.3)	77/496 (15.5)	0.001
Orthostatic hypotension (%)	127 (20.1)	39/133 (29.3)	88/496 (17.7)	0.003
Eye diseases (%)	191 (30.4)	44/133 (33.1)	147/496 (29.6)	0.458
Paralysis (%)	32 (5.1)	16/133 (12)	15/496 (3)	< 0.001
Amputation (%)	26 (4.1)	8/133 (6)	18/496 (3.6)	0.223
Dementia (%)	102 (16.2)	35/133 (26.3)	68/496 (13.7)	0.001
**ESRD origin**				
Diabetes mellitus (%)	237 (37.6)	48/133 (36.1)	189/496 (38.1)	0.688
Nephrosclerosis (%)	162 (25.8)	37/133 (27.8)	125/496 (25.2)	0.577
Chronic glomerulonephritis (%)	57 (9)	14/133 (10.5)	43/496 (8.7)	0.499
Peripheral artery diseases (%)	128 (20.3)	30/133 (22.6)	98/496 (19.8)	0.469
**Prescription**				
Antiparkinsonian drugs (%)	6 (0.9)	3/133 (2.3)	3/496 (0.6)	0.112
Hypnotics (%)	151 (24)	42/133 (31.6)	109/496 (22)	0.029
Vasopressor drugs (%)	133 (21.1)	39/133 (29.3)	94/496 (19)	0.012
Antihypertensive drugs (%)	421 (66.9)	89/133 (66.9)	332/496 (66.9)	*p* > 0.99
Psychotropic drugs (%)	29 (4.6)	6/133 (4.5)	23/496 (4.6)	*p* > 0.99

Table 1 presents the baseline characteristics of the patients. Patients in the fall group were older than those in the non-fall group (73.8 years vs. 67.2 years, *p* < 0.001). The heights of those in the fall group were found to be lower than those in the non-fall group (159.4 cm vs. 161.9 cm, *p* = 0.006).The most common complications in the fall group were orthopedic diseases (49.6% vs. 26.8%, *p* < 0.001), cerebrovascular diseases (29.3% vs. 15.5%, *p* = 0.001), orthostatic hypotension (29.3% vs. 17.7%, *p* = 0.003), paralysis (12.0% vs. 3.0%, *p* < 0.001), and dementia (26.3% vs. 13.7%, *p* = 0.001). *DW* desired weight, *BMI* body mass index

### Investigation of falls within the dialysis center

The data were examined retrospectively from medical records; falls were defined as the incidents that occurred in the dialysis room between April 1, 2016, and May 31, 2018; dialysis room was defined as HD bed, waiting room, changing room, corridor, not including spaces outside the facility or shuttle cars. Patients were categorized into the non-fall (*n* = 496) and fall groups (*n* = 133). A total of 106 items were investigated as causes of falls. Table 1 represents patient characteristics including demographic data, complications, end-stage renal failure origin, and prescription in the fall and non-fall groups. Table 2 shows.

**Table 2:** Activities of daily living and dialysis characteristics

	Non-falls (*n* = 496)	Falls (*n* = 133)	*P*-value
**Walking situation**			
Wheelchair (%)	46/496 (9.3)	46/133 (34.6)	< 0.001
Movement assist (%)	74/496 (14.9)	58/133 (43.6)	< 0.001
Walking aid (%)	58/496 (11.7)	52/133	< 0.001
**Footwear in the clinic**			
Slippers (%)	440/496 (88.7)	110/133 (82.7)	0.076
Sandals (%)	24/496 (4.8)	10/133 (7.5)	0.278
Shoes (%)	14/496 (2.8)	13/133 (9.8)	0.001
**Character in home**			
Smoking habits (%)	51/496 (10.3)	11/133 (8.3)	0.623
Patient’s compliance (%)	71/493 (14.4)	25/133 (18.8)	0.223
Patient’s family compliance (%)	7/491 (1.4)	3/133 (2.3)	0.451
**Care service**			
Home-visit rehabilitation (%)	14/496 (2.8)	14/133 (10.5)	< 0.001
Primary nursing care requiement authorization (y/n)	108/496 (21.8)	72/133 (54.1)	< 0.001
Day Service	13/496 (2.6)	11/133 (8.3)	0.003
Day Care	17/496 (3.4)	16/133 (12)	< 0.001
**Dialysis method**			
HDF (%)	93/496 (18.8)	28/133 (21.1)	0.538
HD (%)	315/496 (63.5)	73/133 (54.9)	0.072
IHDF (%)	90/496 (18.1)	33/133 (24.8)	0.109
Dialysis time (h)	3.4 (1.3)	3.4 (1.4)	0.685
Dialysis duration (y)	67.15 (68.11)	78.1( 82.1)	0.107
**Vascular access (%)**			
Left side of the body (%)	312/496 (62.9)	74/133 (55.6)	0.133
Upper body (%)	426/496 (85.9)	109/133 (82)	0.274

Table 2 presents data regarding activities of daily living and dialysis characteristics. In terms of the walking situation, several in the fall group were wheelchair users (34.6% vs. 9.3%, *p* < 0.001), required walking assistance (43.6% vs. 14.9%, *p* < 0.001), and were walking-aid users (39.1% vs. 11.7%, *p* < 0.001). Regarding footwear, more shoe users (9.8% vs. 2%, *p* = 0.001) were found in the fall group. Additionally, for care service items, visiting care (8.3% vs. 2.6%, *p* = 0.008), outpatient rehabilitation (12.0% vs. 3.4%, *p* < 0.001), and home-visit rehabilitation (10.5% vs. 2.8%, *p* < 0.001) were more common in the fall group. *HD* hemodialysis, *HDF* hemodiafiltration, *IHDF* intermittent infusion hemodiafiltration; Day Service, Senior Day Care Centers where seniors can engage with one another through various activities; Day Care, Adult Day Health Care Centers where seniors can get the specialized care they need, such as physical and occupational therapy

ADL including walking outside the facility, footwear, character in the home, care service, dialysis method, and dialysis vascular access. Table 3 presents laboratory data of the two groups. The injuries and illnesses were defined from the medical record information according to the ICD-10 codes [29], including orthopedic diseases (ICD-10 M00-M99). Walking aid was defined as using aids such as a walker or cane and holding onto an object for support [1]. In addition to the physical functions, we examined data such as blood test reports, presence of complications, nursing care status, and social background as possible risk factors for falls. In Japan, to use the nursing service provided by nurse-care insurance, the necessity of nursing has to be recognized (nurse-care requirement authorization).

**Table 3:** Laboratory data in each group

	Falls (*n* = 133)	Non-falls (*n* = 496)	
	mean ± SD	mean ± SD	*p*
Pre-BUN (mg/dL)	60.9 ± 11	61.8 ± 11.5	0.414
Post-BUN (mg/dL)	18.5 ± 4.9	19.2 ± 5.1	0.217
Creatine (mg/dL)	9.3 ± 2.3	10.2 ± 2.6	< 0.001
Pre-eGFR (mL/分 /1.73 m^2^)	4.9 ± 2.1	4.6 ± 1.8	0.098
Post-eGFR (mL/分/1.73 m^2^)	14.5 ± 5.4	13.5 ± 4.9	0.05
Pre-UA (mg/dL)	6.9 ± 1.2	6.9 ± 1.3	0.779
Post-UA (mg/dL)	2 ± 0.5	1.9 ± 0.5	0.281
Pre-Na (mEq/L)	138.2 ± 2.7	138.6 ± 2.4	0.08
Post-Na (mEq/L)	138.4 ± 1.5	138.3 ± 1.1	0.665
Pre-K (mEq/L)	4.7 ± 0.6	4.7 ± 0.6	0.912
Post-K (mEq/L)	3.3 ± 0.2	3.3 ± 0.3	0.912
Cl (mEq/L)	103.2 ± 2.9	103.7 ± 2.9	0.125
Pre-Ca (mg/dL)	8.7 ± 0.6	8.7 ± 0.5	0.946
Post-Ca (mg/dL)	9.7 ± 0.5	9.7 ± 0.4	0.128
P (mg/dL)	5.2 ± 0.8	5.2 ± 0.8	0.355
Mg (mg/dL)	2.4 ± 0.3	2.5 ± 0.3	0.021
TP (g/dL)	7 ± 0.4	7 ± 0.4	0.834
Alb (g/dL)	3.5 ± 0.3	3.6 ± 0.3	0.002
T-cho (mg/dL)	157 ± 29	156.7 ± 28.6	0.927
TG (mg/dL)	101.6 ± 41.3	122.2 ± 80.6	0.003
HDL-C (mg/dL)	52 ± 13.5	50.3 ± 80.6	0.23
LDL-C (mg/dL)	83.8 ± 23.8	82.6 ± 22.6	0.601
GA (%)	19.9 ± 5.4	19.5 ± 4.2	0.512
Glu (mg/dL)	142.1 ± 36.2	134.5 ± 37.2	0.035
AST (U/L)	16.8 ± 7.7	15.2 ± 6.2	0.021
ALT (U/L)	10.4 ± 6.4	10.2 ± 5.6	0.774
LDH (U/L)	203.5 ± 36.2	193.7 ± 34.6	0.005
ALP (U/L)	278.9 ± 93.4	259.5 ± 104.6	0.056
γ-GTP (U/L)	26.5 ± 22.2	27 ± 28.2	0.858
ChE (U/L)	217.7 ± 56.1	232.1 ± 58.9	0.012
T-Bil (mg/dL)	0.3 ± 0.1	0.3 ± 0.1	0.912
Amy (U/L)	126.6 ± 48.7	129.7 ± 55.2	0.565
Fe (μg/dL)	66.3 ± 11.9	70.8 ± 17	0.005
UIBC (μg/dL)	195.2 ± 39	200.9 ± 43.2	0.167
Fer (ng/mL)	86 ± 72.7	78.6 ± 59.7	0.231
CPK (IU/L)	120.6 ± 158.9	112.4 ± 88.8	0.817
CPK-MB (ng/mL)	13.3 ± 2.9	17 ± 7.1	0.37
CRP (mg/dL)	0.7 ± 0.7	0.5 ± 0.9	0.063
Adjusted calcium (mg/dL)	9.2 ± 0.5	9.1 ± 0.5	0.103
TSAT(%)	26.2 ± 5.5	26.9 ± 7.2	0.288
β2MG-S	27.9 ± 5.8	27.6 ± 6.2	0.581
I-PTH (pg/mL)	202.9 ± 106.1	223 ± 143.9	0.135
HANP (pg/mL)	172.3 ± 189.1	114 ± 158.8	0.015
BNP (pg/mL)	470.4 ± 624.1	290.2 ± 464.5	0.045
WBC (10^2^/μL)	63.4 ± 15.4	64 ± 17.1	0.716
RBC (10^4^/μL)	355.9 ± 27.8	360.4 ± 29.3	0.111
Hb (g/dL)	10.8 ± 0.5	10.9 ± 0.5	0.119
Ht (%)	33.8 ± 1.6	33.9 ± 1.5	0.408
HCV	95.4 ± 5.3	94.6 ± 5.7	0.139
MCH (fL)	30.6 ± 1.8	30.5 ± 2.1	0.448
MCHC (pg)	32.1 ± 0.4	32.2 ± 0.5	0.024
Plt (10^2^/μL)	18.2 ± 5.6	18.8 ± 5.7	0.224
RET (%)	14.9 ± 5	15.2 ± 5.1	0.663
Basophils (%)	0.6 ± 0.2	0.6 ± 0.2	0.39
Eosinophils (%)	4.8 ± 2.6	4.7 ± 3.6	0.716
Neutrophils (%)	67.3 ± 6.6	66.1 ± 7.1	0.074
Lymphocytes (%)	19.2 ± 6.2	20.6 ± 5.8	0.015
Monocytes (%)	8.1 ± 1.9	8 ± 2	0.637
Systolic Bp Start (mmHg)	157.8 ± 18.2	154.7 ± 18.7	0.092
Diastolic Bp Start (mmHg)	77.3 ± 10.9	79.2 ± 11	0.082
Systolic Bp End (mmHg)	146 ± 15.1	144.4 ± 15.8	0.298
Diastolic Bp End (mmHg)	74.5 ± 9.1	76.8 ± 9.9	0.015
HR Start (bpm)	73.2 ± 9.3	75.4 ± 10.4	0.026
HR End (bpm)	71 ± 9.9	72.4 ± 11	0.197

Table 3 presents data on blood tests. Patients in the fall group had significantly lower pre-dialysis levels of creatinine (9.3 mg/dL vs. 10.2 mg/dL *p* < 0.001), albumin (3.5 g/dL vs. 3.6 g/dL, *p* = 0.002), triglyceride (101.6 mg/dL vs. 122.2 mg/dL, *p* = 0.003), and iron (66.3 μg/dL vs. 70.8 μg/dL *p* = 0.005) than those in the non-fall group. Lactate dehydrogenase (LDH) levels were higher in the fall group (203.5 U/L vs. 193.7 U/L, *p* = 0.005)

### Statistical analysis

All statistical analyses were performed using SAS® (SAS, Cary, NY, USA). Each independent variable was evaluated for its association with falls. Unpaired t-test was used to evaluate the fall and non-fall examination results. Based on a known association with falls or clinical judgment, multivariate analysis was performed on covariates that were significantly correlated in the univariate analysis (*p* < 0.05).

Univariate logistic regression was performed for each demographic data (Table 1), ADL data (Table 2), and laboratory data (Table 3) for accidental falls. Multivariate logistic regression was then conducted. Covariates were used based on their correlation with accidental falls, as outlined in Table 4. Covariates were included in the multivariate analysis if the probability was ≤ 0.001. The results indicated that the multivariate analysis, which used covariates with a *p* ≤ 0.001, was effective in predicting accidental falls.

**Table 4:** Univariate logistic regression for accidental fall by each covariate from Tables 1, 2, and 3

Covariates	B	SE	*p*	Exp(B)	95%C.I. Lower	Upper
Age_Base	0.048	0.009	< 0.001	1.049	1.030	1.068
Gender	-0.244	0.202	0.226	0.784	0.528	1.163
Hight	-0.029	0.010	0.006	0.972	0.952	0.992
BMI	-0.033	0.026	0.198	0.967	0.919	1.018
Living with of without family or unknown	0.147	0.141	0.296	1.158	0.879	1.526
BUN (mg/dL)	-0.007	0.009	0.414	0.993	0.976	1.010
Crea (mg/dL)	-0.148	0.040	< 0.001	0.863	0.798	0.932
eGFR (ml/min)	0.078	0.047	0.098	1.081	0.986	1.186
UA (mg/dL)	-0.021	0.076	0.779	0.979	0.844	1.136
Na (mEq / L)	-0.069	0.039	0.080	0.933	0.864	1.008
K (mEq / L)	0.018	0.165	0.912	1.018	0.737	1.408
Cl (mEq / L)	-0.051	0.033	0.125	0.950	0.890	1.014
Ca (mg/dL)	0.013	0.186	0.946	1.013	0.703	1.459
P (mg/dL)	-0.108	0.117	0.355	0.897	0.713	1.129
Alb	-1.094	0.353	0.002	0.335	0.168	0.669
T-cho	0.000	0.003	0.927	1.000	0.994	1.007
TG	-0.006	0.002	0.003	0.994	0.990	0.998
HDL-C	0.008	0.006	0.230	1.008	0.995	1.020
LDL-C	0.002	0.004	0.601	1.002	0.994	1.011
Glu	0.005	0.003	0.035	1.005	1.000	1.010
AST	0.031	0.014	0.021	1.032	1.005	1.059
ALT	0.005	0.017	0.774	1.005	0.972	1.038
LDH	0.007	0.003	0.005	1.007	1.002	1.013
ALP	0.002	0.001	0.056	1.002	1.000	1.003
γ-GTP	-0.001	0.004	0.858	0.999	0.992	1.007
ChE	-0.005	0.002	0.012	0.995	0.992	0.999
T-Bil	0.086	0.773	0.912	1.090	0.239	4.961
Amy	-0.001	0.002	0.565	0.999	0.995	1.003
Fe	-0.019	0.007	0.005	0.981	0.968	0.994
UIBC	-0.003	0.002	0.167	0.997	0.992	1.001
TIBC	-0.007	0.003	0.010	0.993	0.988	0.998
Fer	0.002	0.001	0.231	1.002	0.999	1.005
CPK	0.001	0.003	0.817	1.001	0.995	1.007
Ca(adjusted)	0.301	0.185	0.103	1.351	0.941	1.940
TSAT	-0.016	0.015	0.288	0.984	0.956	1.013
β2MG-S	0.009	0.016	0.581	1.009	0.978	1.041
I-PTH	-0.001	0.001	0.135	0.999	0.997	1.000
HANP	0.002	0.001	0.015	1.002	1.000	1.003
BNP	0.001	0.000	0.045	1.001	1.000	1.001
WBC	-0.002	0.006	0.716	0.998	0.986	1.009
RBC	-0.006	0.003	0.111	0.994	0.988	1.001
Hb	-0.317	0.203	0.119	0.728	0.489	1.085
Ht	-0.054	0.065	0.408	0.947	0.833	1.077
HCV	0.026	0.018	0.139	1.027	0.991	1.063
Plt	-0.022	0.018	0.224	0.979	0.945	1.013
Ret	-0.009	0.020	0.663	0.992	0.954	1.030
sBp start	0.009	0.005	0.092	1.009	0.999	1.020
dBp start	-0.016	0.009	0.082	0.984	0.967	1.002
sBp end	0.007	0.006	0.298	1.007	0.994	1.019
sBp end	-0.025	0.010	0.015	0.975	0.956	0.995
H.R. start	-0.022	0.010	0.026	0.978	0.960	0.997
H.R. end	-0.012	0.009	0.197	0.988	0.970	1.006
Blood Access Left side of the body (%)d 1	-0.302	0.198	0.127	0.740	0.502	1.090
Blood Access Upper Extremety 1	-0.293	0.260	0.260	0.746	0.449	1.242
HDF	0.145	0.242	0.550	1.156	0.719	1.856
HD	-0.358	0.198	0.070	0.699	0.475	1.030
IHDF	0.398	0.232	0.086	1.489	0.945	2.346
Dialysis time	-0.030	0.073	0.685	0.971	0.842	1.120
Dialysis Period	0.002	0.001	0.110	1.002	1.000	1.005
Primary nursing care requiement authorization(a 5-level graded system under health insurance	1.445	0.205	< 0.001	4.240	2.836	6.340
Day Care Utilization	1.349	0.363	< 0.001	3.853	1.891	7.853
Day Service Utilization	1.209	0.422	0.004	3.35	1.465	7.661
Home Visit Rehabilitation	1.399	0.392	< 0.001	4.050	1.880	8.726
DM:1	-0.086	0.203	0.670	0.917	0.616	1.365
Nephrosclerosis	0.134	0.219	0.540	1.144	0.744	1.759
Chronic Glomerlar Nephritis	0.215	0.324	0.508	1.239	0.656	2.341
PAD1	0.168	0.236	0.477	1.183	0.745	1.879
Orthopedic Disease	0.989	0.201	< 0.001	2.689	1.814	3.986
Cardiovascular Disease	0.320	0.196	0.103	1.378	0.937	2.025
Cerebrovascular Disease	0.814	0.227	< 0.001	2.258	1.446	3.525
Orthostatic Hypotension	0.654	0.224	0.003	1.924	1.241	2.983
Cataract and Retinopathy and Blindness	0.160	0.209	0.443	1.174	0.779	1.768
Paralysis	1.478	0.374	< 0.001	4.385	2.107	9.125
Limb amputation	0.530	0.437	0.224	1.700	0.722	3.999
Wheelchair in transportation vehicle or dialysis room	1.643	0.239	< 0.001	5.172	3.237	8.265
Assistant During Transportation	1.484	0.216	< 0.001	4.410	2.891	6.728
Walking Aids	1.579	0.226	< 0.001	4.848	3.113	7.551
Prosthetic Devise ( Artificial leg or Corset)	1.163	0.461	0.012	3.200	1.298	7.893
Antiparkinson Agent	1.333	0.822	0.105	3.792	0.757	19.009
Sleeping Pills	0.494	0.216	0.022	1.639	1.074	2.501
Pressure Boosting Agents	0.573	0.222	0.010	1.774	1.148	2.743
Antihypertensive Agents	-0.001	0.208	0.997	0.999	0.665	1.501
Psychotropic Drug	-0.029	0.469	0.951	0.972	0.387	2.437
Dementia	0.810	0.236	0.001	2.248	1.415	3.572
Slipper	-0.496	0.270	0.066	0.609	0.359	1.033
Sandal	0.469	0.390	0.229	1.599	0.745	3.432
Shoes in the dialysis room	1.316	0.398	0.001	3.730	1.708	8.144
Smoking Habit	-0.240	0.348	0.490	0.787	0.398	1.555
Poor Compliance of indivisual	0.319	0.256	0.213	1.376	0.832	2.274
Poor Compliance of family member	0.467	0.697	0.503	1.596	0.407	6.256

Table 4 presents the univariate analyses of each of the three groups of covariates that correlated with falls in the univariate logistic regression. In demographic data and complications, orthopedic diseases, cerebrovascular diseases, orthostatic hypotension, paralysis, cognitive dysfunction, and hypnotic prescription were correlated with accidental falls. In the group of ADL, the use of wheelchairs and walking aids outside of the facility was correlated with accidental falls. In the univariate analysis, wheelchair use was negatively correlated with falls (EXP(B) 0.24). In the group of laboratory data, creatinine level (mg/dL), albumin level (g/dL), and systolic pressure (mmHg) were correlated with falls. Serum creatinine and albumin levels were negatively correlated with falls

Covariates used in each group for multivariate logistic regressions were significantly correlated with falls in each univariate analysis. Statistical significance was set at *p* < 0.05.

Among the 629 hemodialysis patients, there were no instances of missing data.

## Results

During the study period, 133 patients experienced falls. Table 1 presents the baseline characteristics of the patients. Patients in the fall group were older than those in the non-fall group (73.8 years vs. 67.2 years, *p* < 0.001). The heights of those in the fall group were found to be lower than those of patients in the non-fall group (159.4 cm vs. 161.9 cm, *p* = 0.006).

The most common complications in the fall group were orthopedic diseases (49.6% vs. 26.8%, *p* < 0.001), cerebrovascular diseases (29.3% vs. 15.5%, *p* = 0.001), orthostatic hypotension (29.3% vs. 17.7%, *p* = 0.003), paralysis (12.0% vs. 3.0%, *p* < 0.001), and dementia (26.3% vs. 13.7%, *p* = 0.001).

Table 2 shows data regarding ADL and dialysis characteristics. In terms of walking situation, several patients in the fall group were wheelchair users (34.6% vs. 9.3%, *p* < 0.001), required walking assistance (43.6% vs. 14.9%, *p* < 0.001), and were walking-aid users (39.1% vs. 11.7%, *p* < 0.001). Regarding footwear, more shoe users (9.8% vs. 2%, p = 0.001) were found in the fall group. Moreover, for care service items, visiting care (8.3% vs. 2.6%, *p* = 0.008), outpatient rehabilitation (12.0% vs. 3.4%, *p* < 0.001), and home-visit rehabilitation (10.5% vs. 2.8%, *p* < 0.001) were more common in the fall group.

Table 3 presents data on blood tests. Patients in the fall group had significantly lower pre-dialysis levels of creatinine (9.3 mg/dL vs. 10.2 mg/dL *p* < 0.001), albumin (3.5 g/dL vs. 3.6 g/dL, *p* = 0.002), triglycerides (101.6 mg/dL vs. 122.2 mg/dL, *p* = 0.003), and iron (66.3 μg/dL vs. 70.8 μg/dL *p* = 0.005) than those in the non-fall group. Lactate dehydrogenase (LDH) levels were higher in the fall group (203.5 U/L vs. 193.7 U/L, *p* = 0.005).

Table 4 reveals the univariate analyses of each of the three groups of covariates that correlated with falls in the univariate logistic regression. In the demographic data and complications, orthopedic diseases, cerebrovascular diseases, orthostatic hypotension, paralysis, cognitive dysfunction, and hypnotic prescription were correlated with accidental falls (*p* < 0.05). In the ADL group, the use of wheelchairs and walking aids outside of the facility was correlated with accidental falls (EXP(B) 3.31; *p* < 0.001). In the univariate analysis, wheelchair usage negatively correlated with falls (EXP(B) 0.24; *p* < 0.05). Laboratory data indicated that creatinine level (mg/dL), albumin level (g/dL), and systolic blood pressure (mmHg) were correlated with falls. Serum creatinine and albumin levels were negatively correlated with falls.

Table 5 presents the multivariate analyses of three models for the outcome of accidetal fall. Covariates that were correlated with accidental falls and had probabilities ≤ 0.001, as presented in Table 4, were used in the analyses. The results indicated that multivariate analysis, which utilized covariates from Table 4, revealed significant correlations between falls and the use of a walking aid, orthopedic diseases, cerebrovascular disease and age. These covariates were also significantly correlated with falls in the univariate analyses (Table 4).

**Table 5:** Multivariate logistic regression

Covariates	β	S.E	*p*	Exp(B)	EXP(B) 95% C.I. Lower	Upper
Age	0.025	0.012	0.032	1.025	1.002	1.048
Wheelchair in transportation vehicle or dialysis room	0.711	0.378	0.060	2.037	0.971	4.274
Walking Aids	1.143	0.274	< 0.001	3.135	1.833	5.364
Assistant During Transportation	0.169	0.366	0.644	1.184	0.578	2.429
Paralysis	0.554	0.453	0.222	1.741	0.716	4.233
Primary nursing care requiement authorization(a 5-level graded system under health insurance	0.104	0.299	0.728	1.110	0.617	1.995
Home Visit Rehabilitation	0.719	0.454	0.113	2.053	0.843	4.995
Day Care Utilization	0.650	0.439	0.138	1.916	0.811	4.526
Orthopedic Disease	0.825	0.227	< 0.001	2.283	1.462	3.565
Cerebrovascular Disease	0.580	0.270	0.032	1.786	1.052	3.031
Serum Creatinine (mg/dL)	0.006	0.051	0.911	1.006	0.910	1.112
Shoes in the dialysis room	0.036	0.473	0.939	1.037	0.410	2.619
Dementia	0.096	0.310	0.758	1.100	0.599	2.020

Table 5 presents the multivariate analyses for the outcomes of accidental falls. Covariates were selected based on their correlation with accidental falls in Table 4, and were included if their probabilities were ≤ 0.001. Multivariate analysis utilized covariates with *p* ≤ 0.001, which included age, wheelchair in transportation vehicle or dialysis room, walking aids, assistant during transportation, paralysis, primary nursing care requirement authorization, home visit rehabilitation, daycare utilization, orthopedic disease, cerebrovascular disease, serum creatinine (mg/dl), shoes in the dialysis room, and dementia. The multivariate logistic analysis revealed that age, use of a walking aid, and orthopedic diseases and cerebrovascular disease were significantly correlated with falls among all the covariates with *p* ≤ 0.001 in the univariate analysis (Table 4)

## Discussion

### Fall factors

This study showed that walking aid users and patients with orthopedic disease complications had a higher risk of falling. We defined orthopedic diseases according to ICD10 codes S00-S99 and T07-T88, which include injuries to extremities and unspecified effects of external causes. Data were obtained from claims records. Generally, prior falls, visual impairment, urinary incontinence, and functional limitations have been identified as the strongest predictors of recurrent falls [30]. Moreover, the study participants were 65 years or older. Among dialysis patients, despite numerous demographic, ADL, and laboratory data covariates, older patients who required walking aids were found to be at a high risk for falls. Walking-aid users are at a high risk because of disease complications, causing muscle weakness and a decline in physical function. Using a walking aid may reflect the deterioration of physical function, thus increasing the risk of falling. Moreover, some studies have suggested that using walking aids is a risk factor for future falls among a non-disabled older population living in residential settings [31]. Therefore, the use of walking aids, especially in dialysis patients, should be assessed immediately at the first visit.

Furthermore, orthopedic diseases cause a decline in physical function. In dialysis patients, the skeletal system is affected by dialysis amyloidosis, and the risk of complications increases as the dialysis period is extended. Thus, dialysis patients should be screened early for this disease.

Risk factors can be extrinsic (external to the individual) or intrinsic (within the person) [21, 32]. Based on the results of the study, the use of walking aids and complications of orthopedic diseases were believed to be the intrinsic factors (affecting physical performance, such as muscle strength and balance).

Muscle strength and balance were not affected by the manpower required to secure safety and the time required for the examination. However, the results of this study did not require a special examination. Some of the items, such as muscle strength, could be evaluated without waiting for the results of the initial test and imposing a burden on the patient because it was possible to quickly understand the items that could be caused by the intrinsic factors that increase the fall risk.

### Significance of early screening

According to our results, a patient should be screened for orthopedic diseases, and walking-aid users should be carefully monitored at the start of hospital visits based on medical record information and appearance as they are at an heightened risk of falls.

### Need for early fall protection

The risk of falls should be reduced in patients by conducting a risk screening at the start of hospital visits, taking preventive measures, and establishing a safe environment. Environmental improvement is a measure that allows for immediate action and is considered effective in early fall prevention. To identify risk factors for falls, older patients discharged from the internal medicine ward, particularly those with short admission durations, should be carefully assessed. Integration into a fall prevention program should be considered for high-risk patients [26].

A substantial proportion of the relationship between walking aids and future falls can be explained by an altered spatiotemporal gait pattern, increased age, and psychotropic drug intake [31, 33]. It is considered necessary for patients with walking aids to have a re-examination of oral medication and check for spatiotemporal walking patterns.

For patients with altered spatiotemporal gait patterns, a professional evaluation of the environment where the assistive device is used and the physical function of the patient who uses the assistive device should be conducted. Moreover, consultation with a specialist, such as a physical therapist, is considered effective.

The concept of renal rehabilitation (RR) has been proposed in Japan [34]. RR includes coordinated, multifaceted interventions designed to optimize a renal patient’s physical, psychological, and social functioning, in addition to stabilizing, slowing, or even reversing the progression of renal deterioration, thereby reducing morbidity and mortality. RR has five major components: exercise training, diet and fluid management, medication and medical surveillance, education, and psychological and vocational counseling.

Comprehensive team efforts are expected to be effective in early fall prevention. Older people, orthopedic patients, and dialysis patients who use walking aids require caution in daily care and examination of appropriate methods of care and hospital visits. Therefore, regular reviews are necessary.

Moreover, reducing the risk of falls as a long-term measure by incorporating exercise therapy to prevent physical deterioration is warranted. Decreased muscle mass and weakness are independent factors of ADL and instrumental ADL reduction [35, 36]. Exercise therapy is crucial for the long-term prevention of ADL reduction and reduction of fall risk.

Interventions with walking aids should be set not only by using the equipment but also by considering the environment and factors relating to the patient. Therefore, it is critical to evaluate whether the walking aid is suitable for the patient, select an appropriate tool, educate the user on its proper use, and improve the environment. That is, extrinsic risk interventions are required. It is crucial to perform extrinsic risk interventions early from the baseline and start long-term efforts to address the intrinsic factors.

The main limitation of this study was that outcomes were based on the number of falls in the facility room and not in the patient’s home. Nevertheless, the method for preventing falls in the dialysis room can be applied to all patients, including the older patients. Additionally, wheelchair usage outside the facility negatively correlated with falls in the univariate logistic regression analysis (Table 4, ADL). This could mean that using a wheelchair outside may prevent falls; however, in the multivariate analysis (Table 5, model 2), wheelchair usage was a risk factor (Exp(B) 2.245) unlike other covariates.

To prevent dialysis patients from falling in a dialysis center, exercise therapy to reduce the long-term risk of falls should be started. However, to obtain a long-term effect, it is critical to detect high-risk patients at the early stage of the outpatient visit and create a safe environment, such as setting appropriate walking aids to prevent short-term falls.

## Conclusion

In the dialysis clinic, patients who use walking aids and have complicated orthopedic or cerebrovascular diseases are at a heightened risk of falls in the dialysis room. Therefore, creating a safe environment for these patients may help prevent falls. These findings have the potential to prevent falls in various facilities, including hospitals, and are not limited to just dialysis patients.

In the dialysis clinic, early detection of patients using walking aids outside the facility and those with complicated orthopedic and cerebrovascular diseases, and creating a safe environment, such as providing appropriate walking assistance for older patients that do not use walking aids in the dialysis room, can help prevent falls.

## Data Availability

The datasets used and/or analyzed during the current study are available from the corresponding author upon reasonable request.

## Abbreviations

HD: Hemodialysis
CKD: Chronic kidney disease
ADL: Activities of daily living
LDH: Lactate dehydrogenase
RR: Renal rehabilitation
